# Determinants of mortality after hip fracture surgery in Sweden: a registry-based retrospective cohort study

**DOI:** 10.1038/s41598-018-33940-8

**Published:** 2018-10-24

**Authors:** Rasmus Åhman, Pontus Forsberg Siverhall, Johan Snygg, Mats Fredrikson, Gunnar Enlund, Karin Björnström, Michelle S. Chew

**Affiliations:** 1 Department of Anaesthesia and Intensive Care, Department of Medical and Health Sciences, Linköping University, Linköping, S-58185 Sweden; 2 Department of Anaesthesia and Intensive Care, Sahlgrenska University Hospital, 41345 Gothenburg, Sweden; 3 Department of Clinical and Experimental Medicine, Faculty of Medicine and Health, Linköping University, S-58185 Linköping, Sweden; 4 Department of Anaesthesia and Intensive Care, Uppsala University Hospital, 78185 Uppsala, Sweden

## Abstract

Surgery for hip fractures is associated with high mortality and morbidity. The causes of poor outcome are not fully understood and may be related to other factors than the surgery itself. The relative contributions of patient, surgical, anaesthetic and structural factors have seldom been studied together. This study, a retrospective registry-based cohort study of 14 932 patients undergoing hip fracture surgery in Sweden from 1st of January 2014 to 31st of December 2016, aimed to identify important predictors of mortality post-surgery. The independent predictive power of our included variables was examined using Cox proportional hazards modeling with all-cause mortality at longest follow-up as the outcome. Twelve independent variables were considered as interrelated ‘exposures’ and their individual adjusted effect within a single model were evaluated. Kaplan-Meier curves were also generated. Crude mortality rates were 8.2% at 30 days (95% CI 7.7–8.6%) and 23.6% at 365 days (95% CI 22.9–24.2%). Of the 12 factors entered into the Cox regression analysis, age (aHR1.06, p < 0.001), male gender (aHR 1.45, p < 0.001), ASA-PS-class (ASA 1&2 reference; ASA 3 aHR 2.12; ASA 4 aHR 4.79; ASA 5 aHR 12.57 respectively, p < 0.001) and PACU-LOS (aHR 1.01, p < 0.001) were significantly associated with mortality at longest follow-up (up to 3 years). University hospital status was protective (aHR 0.83, p < 0.001) in the same model. Age, gender and ASA-PS-class were strong predictors of mortality after surgery for hip fractures in Sweden. University hospital status and length of stay in the postoperative care unit were also identified as modifiable risk factors after multivariable adjustment and require confirmation in future studies.

## Introduction

Hip fractures are associated with increased mortality, morbidity and financial burden for patients and health care providers^1^. Sweden has one of the highest age-adjusted incidence of hip fractures in the world^2–4^ and the number is predicted to double between 2002 and 2050^5^.

Outcome after hip fracture surgery is likely to be multifactorial. While numerous studies have investigated the association between discrete elements of the perioperative process, such as the impact of surgical delay and after-hours surgery and postoperative outcome, few studies have evaluated the entire perioperative course including patient, surgical, anaesthetic and structural factors. In addition, data on short-term outcomes are often readily available, while longer-term outcomes are less frequently reported.

Previous studies show that patient-related risk factors such as age, male gender and American Society of Anesthesiologists Physical Status (ASA-PS)-class are important determinants of mortality^6–8^. While these factors have consistently been shown to be associated with mortality after hip-fracture surgery, they are not always adjusted for and may confound survival analyses.

The impact of surgical factors such as surgical delay and time in theatre on postoperative outcomes have not been consistently demonstrated^9–14^. For example, some studies suggest a survival benefit with early surgery^9–11^ whilst other data suggest no difference due to surgical delay^12,13^. In a pilot feasibility study, accelerated care consisting of rapid medical optimization and early surgery was associated with decreased postoperative morbidity^14^. Time in theatre is also independently associated with poorer outcomes, however it is highly dependent on the type of surgical procedure undertaken. The type of surgical procedure depends on both fracture localization and the patients’ physical status, thus confounding any survival analysis.

The contribution of anaesthesia and post-anaesthetic care to mortality is also not clear. Large cohort studies and a meta-analysis could not identify differences in mortality due to anaesthetic technique^15–18^ whilst other studies show divergent findings^19,20^. Regarding post-anaesthetic care, Eichenberger *et al*. demonstrated decreased length of stay (LOS) at the post-anaesthesia care unit (PACU) and decreased in-hospital mortality when implementing a clinical pathway in PACU^21^. The effects of PACU-LOS and ICU-admission after hip fracture surgery have been sparingly investigated^22,23^.

Structural factors such as after-hours surgery may be related to adverse outcomes in various types of surgical procedures. Personnel fatigue, decreased availability of staff and equipment have been suggested as factors that could increase mortality. Previous studies investigating after-hours surgery for hip fractures show equivocal results^24–29^. Teaching hospital status is another potentially crucial factor for outcome and has been associated with decreased mortality in studies with careful adjustment for potential confounders^30–32^. However, it is unclear whether this is an intrinsic effect or a reflection of resource availability and surgical delay for non-medical reasons. For example, many rural institutions may require transfer of patients to larger centres for surgical treatment and this may inadvertently cause surgical delay that is deleterious for health^30^. Other studies show a protective effect of teaching hospital status independent of age, sex, comorbidities, surgical delay and complications^32^. At least one study suggests that a non-profit motive may influence the level of in-hospital care^31^, an effect that would be minimized in the Swedish health care system where the vast majority of hospitals are non-profit governmental organizations.

With these limitations in mind, we sought to investigate different aspects of the whole perioperative process. This was done by including structural, patient, anaesthetic, surgical and postoperative care related factors and their potential impact on mortalities in patients undergoing surgery for hip fractures in Sweden. Our intention was to establish an explanatory model with more comprehensive adjustment for risk factors than previous studies. Our hypothesis was that age, gender, ASA-PS-class, surgical delay, time in theatre, type of surgery, after-hours surgery, type of admitting hospital, PACU-LOS and ICU-admission are independently associated with mortality in hip fracture surgery.

## Methods

### Study design

This is a registry-based retrospective cohort study using prospectively collected data from the Swedish PeriOperative Registry (SPOR). Patients ≥18 years of age with a Swedish social security number undergoing surgical hip fracture procedures between 1^st^ of January 2014 and 31^st^ of December 2016 were included. The exposures were a series of patient, surgical, anaesthetic and structural factors in patients subjected to acute hip fracture surgery. All cause-mortality at longest follow-up was our primary outcome. Mortality data was extracted from SPOR and cross-checked with the Swedish Registry of Deaths.

All patients were given information regarding the registry during hospital admission and were given the option to have their data de-registered at any time. Individual patient consent was waived due to the database nature of the study (Regional Ethical Review Board, Gothenburg (nr. 097-17).

Our datafile was extracted the 18^th^ of April 2017.

### Variables

We investigated 12 independent variables that were *a priori* defined. These were age, gender, ASA-PS-class, university hospital status, time of surgery, type of surgery, compliance to surgical urgency planning, surgical delay, time in theatre, type of anaesthesia, PACU-LOS and ICU-admission. These variables were selected based on clinical plausibility and previous findings in the literature, as well as from our own hypotheses where current literature did not provide enough information. These variables were also selected because they had a low proportion of missing data in SPOR and high degree of validity.

### Data collection and cleaning

SPOR was queried for all surgical procedures with procedural codes using the Swedish version of the NOMESCO Classification of Surgical Procedures^33^. We included procedural codes NFJ.XX (surgery for hip fractures) or NFB.XX (hip prosthetic surgery) in combination with a primary diagnosis of hip fractures (diagnosis codes S72.XX). Patients who only had surgical resetting codes (NFJ09 and NFJ19) without secondary surgical procedural codes were excluded. We also excluded patients undergoing elective surgery, and those without mortality data.

SPOR is a prospectively maintained registry with a number of built-in data validation processes. For example, the data are subject to a number of automatic logical controls. Incorrect and/or inconsistent posts are returned to the user for correction prior to inclusion in the database.

Variables were checked for completeness and consistency. Data regarding ASA-PS-class was missing for 799 patients, PACU-LOS for 1310 patients and anaesthetic technique for 231 patients. We could not identify data that were obviously missing in a systematic fashion, therefore all 14932 patients were included for analysis and missing data were imputed.

### Statistical analysis

For the statistical analysis, we used IBM SPSS Statistics (IBM SPSS Statistics for Macintosh, Version 24.0, IBM Corp., Armonk, NY). Descriptive statistics are presented as number of cases, percentages and medians with interquartile ranges (IQR). Mortalities are presented in percentages with 95% Confidence Intervals (CI).

Normality was tested for using the Kolmogorov-Smirnov test. For inter-group differences, the student’s t-test (parametric data) and Mann-Whitney U test (non-parametric data) were used for continuous variables. The χ^2^ test was used for categorical variables.

The independent predictive power of the hospital-, patient-, anaesthesia- and surgery-related factors were examined using survival analyses with Cox proportional hazards modeling with mortality at longest follow-up as the outcome. The 12 independent variables examined were considered to be interrelated ‘exposures’ and their individual adjusted effect within a single model was calculated and presented as adjusted hazard ratios. We used an exploratory model for our multivariable analyses including all plausible variables without taking into account their statistical significance in the univariate analysis. Kaplan-Meier curves were generated.

In order to test whether we fulfilled the assumption of proportional hazards, we assessed Schoenfeld residuals for any variables appearing to violate these assumptions. The proportional hazard assumption was violated (χ2 = 76.5, P < 0.001), however examination of the survival curves over time revealed that the survival probabilities were very similar. We believe that the large number of subjects also influence the probability. Therefore, we proceeded with the Cox regression despite this statistical significance. The robustness of the Cox regression was further tested using multivariable logistic regression analysis, using 30-day and 365-day mortalities as the outcome.

We also performed a sensitivity analysis. To assess the relationship between increasing coverage in the SPOR registry between 2014 and 2016 we split our population in three; all surgeries conducted in 2014, 2015 and 2016. We re-ran the multivariable logistic regression analyses for each year and outcome separately. Further sensitivity analyses were conducted using only full datasets (ie. no imputed values).

## Results

### Baseline characteristics

**Figure 1:**
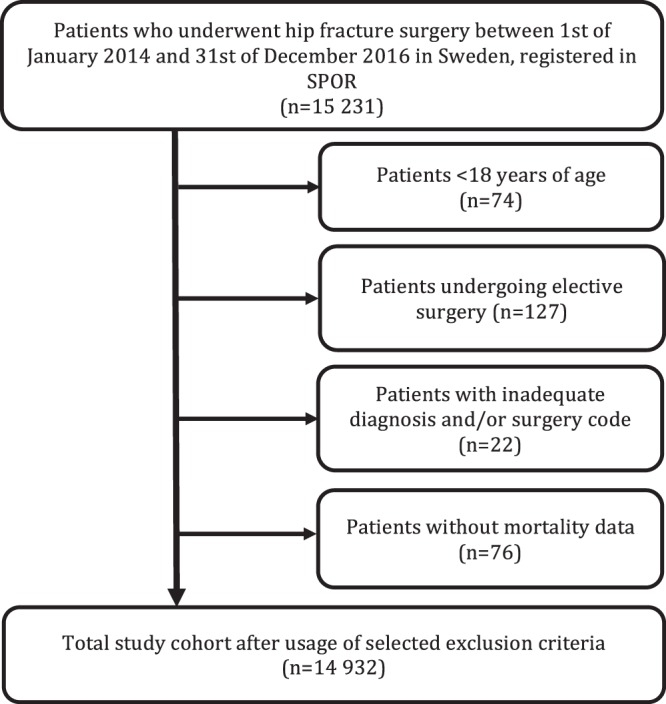
Flowchart demonstrating the inclusion process.

Of the 15231 surgical procedures for hip fractures identified in SPOR, 14 932 were included in this study (Fig. 1). A majority were women (66.8%) and the median age was 83 years. Nearly one-third (64.7%) were aged 80 years or older. Our cohort was unevenly distributed between university and non-university hospitals (16.1% versus 83.9%). ASA-PS-class was also unevenly distributed in the population. More than 50% of all cases had an ASA-score of 3 or more. Only 15 patients were scored as ASA 5.

Out of the five different surgical procedure groups included in the study “Hip replacement: cemented” was the one most commonly occurring in the dataset (32.8%) whereas “Hip replacement: non-cemented” comprised the least number of cases (0.7%). All the remaining three surgical procedure groups included a minimum of 2500 patients. The most common choice of anaesthetic technique in our population was locoregional which comprised more than four fifths of all cases (82.2%).

The median waiting time for surgery was 16.5 hours (IQR 8.4–22.7), where surgical waiting time was defined as the time from electronic registration of the need for surgery (usually at time of diagnosis) to incision. The median time in theatre was 1.1 hours. The median length of stay in the post-anaesthesia care unit (PACU-LOS) was 3.5 hours. After surgery 635 patients (4.3%) were admitted to intensive care.

46.9% of all patients in our cohort underwent surgery during day-time, defined as 0700–1700 Mondays to Thursdays, and 0700–1400 Fridays. Public holidays on week-days were assigned “weekend” status, and ‘weekend’ surgery accounted for 36.1% of all cases. More than seven out of ten surgeries commenced within the planned time limit set by the surgeon (76.9%).

Crude mortality rates were 8.2% at 30 days (95% CI 7.7–8.6%) and 23.6% at 365 days (95% CI 22.9–24.2%), respectively. Table 1 summarizes the characteristics of the cohort included in this study.

**Table 1:** Population characteristics of the study cohort.

	Total (%)
Total number of patients	14932
Median age [IQR]	83 [76–89]
Age groups	
18–54	376 (2.5)
55–69	1575 (10.5)
70–79	3321 (22.2)
80–84	2927 (19.6)
85–89	3511 (23.5)
≥90	3222 (21.6)
Gender: Male	4951 (33.2)
ASA-PS-class (mean)	2.60
1	702 (4.7)
2	5256 (35.2)
3	7118 (47.7)
4	1042 (7.0)
5	15 (0.1)
Type of surgical procedure	
Osteosynthesis: cerclage, spikes, pins	2511 (17.1)
Osteosynthesis: intramedullary nail	4111 (27.9)
Osteosynthesis: screw and plate	3172 (21.6)
Hip replacement: cemented	4820 (32.8)
Hip replacement: non-cemented	102 (0.7)
Anaesthetic technique	
General	2613 (17.8)
Locoregional	12088 (82.2)
Surgical planning	
<0.5 h (emergency)	66 (0.4)
<2 h	98 (0.7)
<6 h	1052 (7.0)
<24 h	12965 (86.8)
>24 h	729 (4.9)
Compliance to surgical planning	11486 (76.9)
Surgical waiting time (hours) [IQR]	16.5 [8.4–22.7]
Time in theatre (hours) [IQR]	1.1 [0.7–1.5]
PACU-LOS (hours) [IQR]	3.5 [2.6–4.7]
Time of surgery	
Day	7004 (46.9)
Other	7928 (53.1)
Weekend	5393 (36.1)
ICU-admission	635 (4.3)
University hospital	2398 (16.1)
30-day mortality	1219 (8.2)
365-day mortality	3517 (23.6)

Absolute numbers (%) are given unless otherwise stated. Time of surgery: Day = 0700–1700 Mondays to Thursdays and 0700–1400 Fridays; Other = all other times than ‘Day’ ie. evenings, nights and weekends; Weekend = Saturdays, Sundays and public holidays.

### Univariable analysis

Nine independent variables were significantly (p ≤ 0.05) associated with 30-day mortality. These were age (p < 0.001), male gender (p < 0.001), ASA-PS-class (p < 0.001), type of surgery (p = 0.031), time in theatre (p = 0.003), surgical waiting time (p < 0.001), compliance to surgical urgency planning (p < 0.001), PACU-LOS (p < 0.001) and ICU-admission post-surgery (p < 0.001).

For 365-day mortality, ten variables were significantly (p ≤ 0.05) associated. These were age (p < 0.001), male gender (p < 0.001), ASA-PS-class (p < 0.001), university hospital (p = 0.001), type of surgery (p < 0.001), time in theatre (p < 0.001), surgical waiting time (p < 0.001), compliance to surgical urgency planning (p < 0.001), PACU-LOS (p < 0.001) and ICU-admission post-surgery (p < 0.001).

### Cox analysis of hazard ratios for mortality at longest follow-up

Results of the Cox regression analysis are shown in Table 2. Of the 12 factors entered into the model, age, male gender, ASA-PS-class, non-university hospital status and PACU-LOS were significantly associated with mortality at longest follow-up (up to 3 years). Each variable was considered as an individual exposure within a single model, and their adjusted hazard ratios after adjusting for the other 11 variables are presented in Table 2.

**Table 2:** Cox regression with mortality at longest follow-up showing adjusted hazard ratios and confidence intervals for the 12 variables entered in the model.

	aHR	95% CI		p-value
Age	1.06	1.05	1.06	<0.001
Male gender	1.45	1.36	1.55	<0.001
ASA-PS-class 1 & 2				Reference
ASA-PS 3	2.12	1.96	2.29	<0.001
ASA-PS 4	4.79	4.30	5.33	<0.001
ASA-PS 5	12.57	6.91	22.85	<0.001
University hospital	0.83	0.76	0.91	<0.001
Time of surgery				
Day				Reference
Evening	1.03	0.93	1.13	0.596
Night	1.17	0.83	1.67	0.368
Weekend	1.03	0.96	1.10	0.455
Type of surgery				
Osteosynthesis: cerclage, spikes, pins				Reference
Osteosynthesis: intramedullary nail	0.97	0.88	1.08	0.596
Osteosynthesis: screw and plate	1.05	0.95	1.17	0.360
Hip replacement: cemented	0.96	0.86	1.07	0.485
Hip replacement: non-cemented	0.64	0.37	1.11	0.114
Compliance to surgical urgency planning	1.00	0.86	1.17	0.971
Surgical waiting time				
<12 h				Reference
12 h–23 h59 min	1.05	0.97	1.13	0.195
>24 h	1.12	0.96	1.32	0.157
Time in theatre	0.96	0.91	1.02	0.214
Type of anaesthesia	1.08	0.99	1.18	0.084
PACU-LOS	1.01	1.00	1.02	<0.001
ICU-admission	1.05	0.85	1.31	0.639

Each variable was considered as an individual exposure and adjusted for the other 11 variables within the same model.

We tested for the robustness of these findings using logistic regression analyses with 30-day and 365-day mortalities as outcomes, adjusting for the same covariates (Supplementary Tables S1–2). For 30-day mortality we found that age (aOR 1.07, p < 0.001); male gender (aOR 1.75, p < 0.001); ASA-PS-class (aOR 2.87 to 20.43, p < 0.001); PACU-LOS (aOR 1.03, p < 0.001) were independently predictive of mortality, whilst university hospital status was protective (aOR 0.81, p = 0.026). Similarly, for 365-day mortality we found the same predictors: age (aOR 1.06, p < 0.001); male gender (aOR 1.02, p < 0.001); ASA-PS-class (aOR 2.66 to 37.26, p < 0.001); PACU-LOS (aOR 1.02, p = 0.001); university hospital status (aOR 0.74, p < 0.001). All logistic regression analyses were repeated with PACU-LOS as a categorical variable (<4, 4–12, >12 h) with similar results.

**Figure 2:**
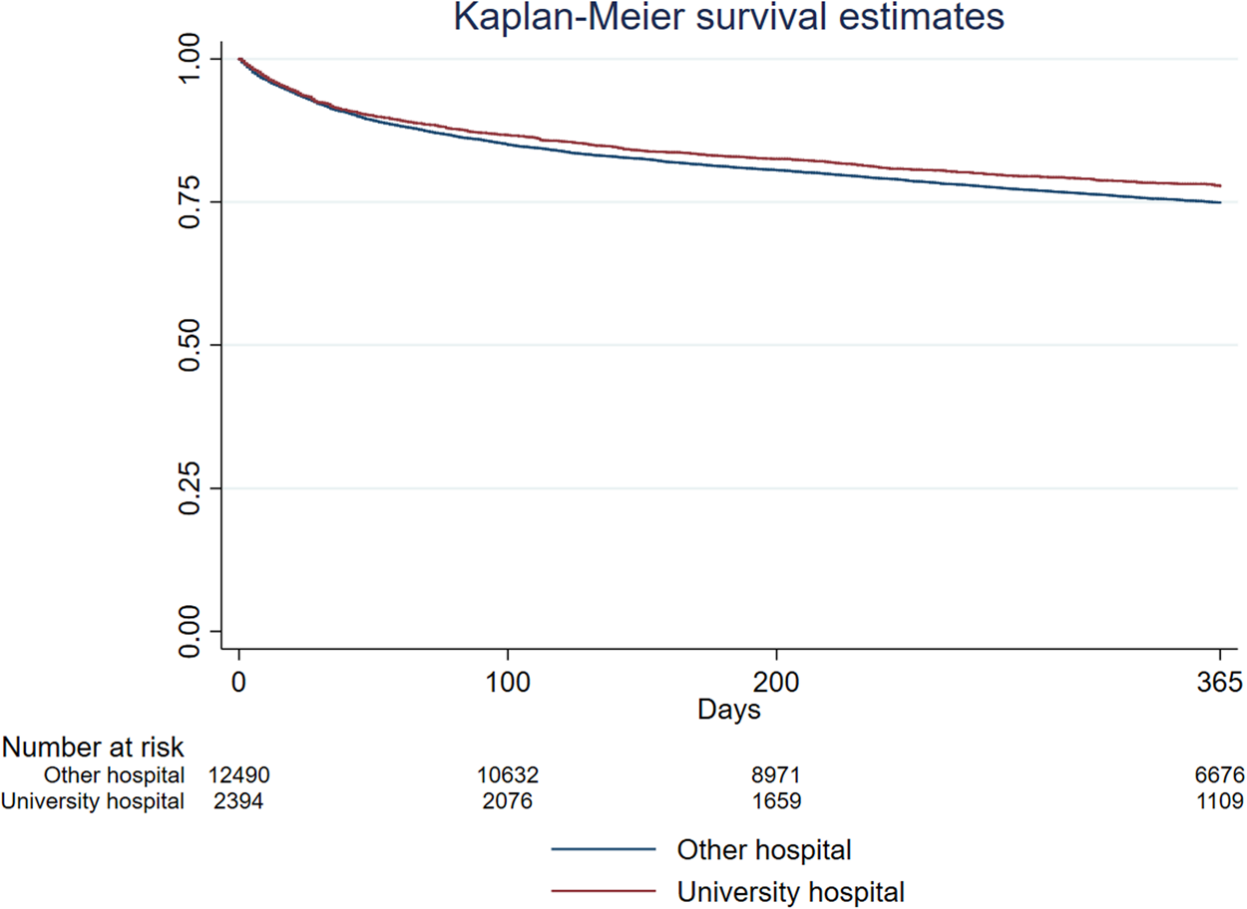
Kaplan Meier Survival Curves illustrating the effect of University Hospital status.

**Figure 3:**
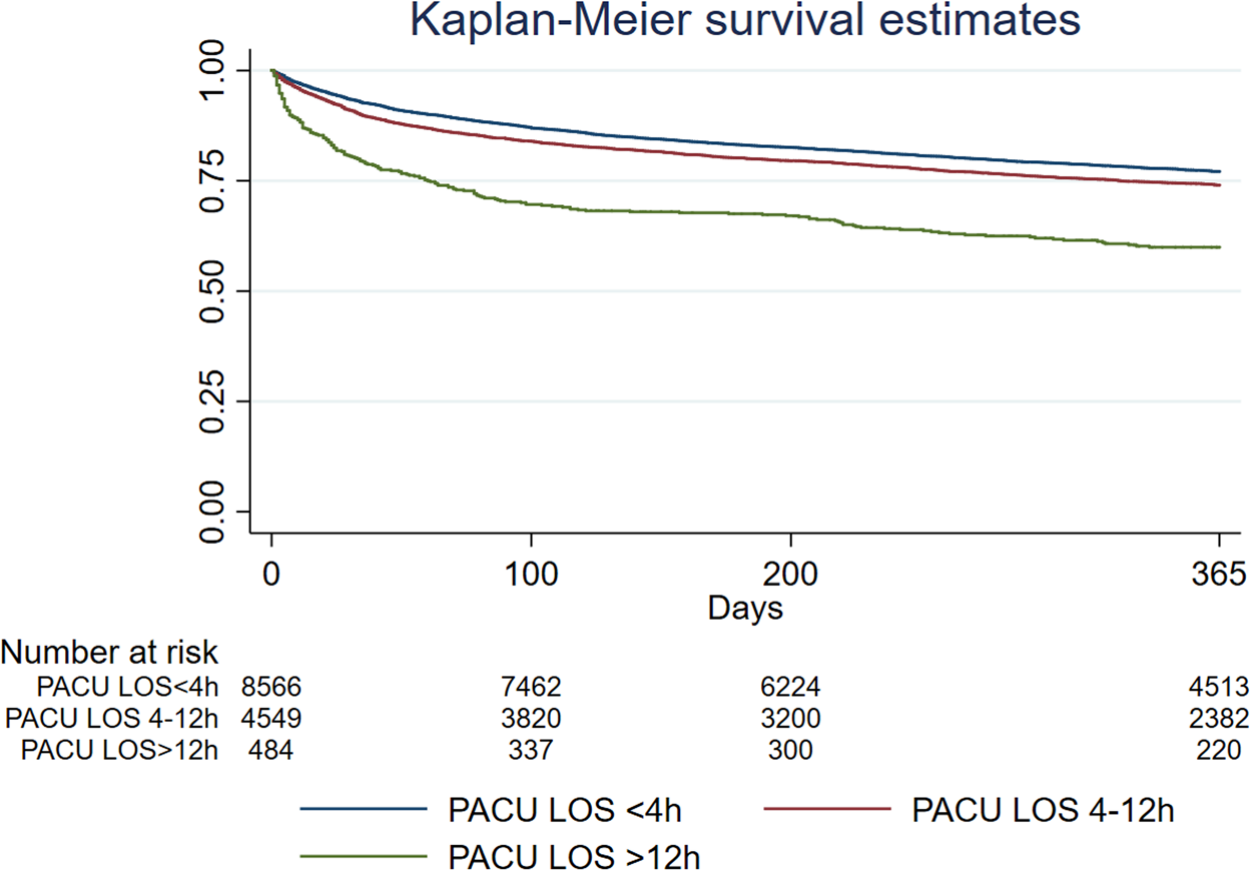
Kaplan Meier Survival Curves illustrating the effect of PACU-LOS.

To illustrate the effect of university hospital status on 365-day mortality we constructed Kaplan-Meier survival curves. When using hospital status as an independent variable, patients undergoing procedures at university hospitals had a statistically significant higher survival compared to non-university hospitals (Fig. 2). Similarly, we also constructed Kaplan-Meier survival curves to illustrate the effect of PACU-LOS on 365-day mortality. When categorized to 0–4 hours, 4–12 hours and 12–24 hours respectively, PACU-LOS was associated with increased mortality in a dose-dependent fashion with highest mortalities seen in patients with length of stays <12 hours (Fig. 3).

Mean ASA-PS-class is higher at university hospitals compared to non-university hospitals (2.70 versus 2.59) despite the higher survival rate.

### Sensitivity analysis

To test for generalizability of results, sensitivity analyses were performed for each calendar year. Our sample size for 2014, 2015 and 2016 were 3202 patients (21.5%), 5188 patients (34.7%) and 6542 patients (43.8%) respectively. The differences may be explained by the fact that SPOR is a newly developed registry and coverage increased over the 3 years.

Both the 30-day and 365-day mortalities were investigated for potential differences between the three calendar years included in the study. Crude mortality rates at 30 days were similar for all three years: 8.4 (CI 7.5–9.4)% vs 8.2 (CI 7.4–8.9)% vs 8.0 (CI 7.4–8.7)% for 2014, 2015 and 2016 respectively (p = NS). At 365 days the crude mortality rates were 24.9 (CI 23.4–26.4)% vs 25.0 (CI 23.9–26.2)% vs 24.2 (CI 22.3–26.1)% for 2014, 2015 and 2016 respectively (p = NS).

To test for the robustness of our primary findings, we re-ran our multivariable logistic regression analyses after subdividing our population into surgeries conducted in 2014, 2015 and 2016. In these multivariable sensitivity analyses, the results were similar to those for the whole population. Age, male gender and ASA-PS-class were significantly associated with mortality in all analyses and with similar odds ratios. The effect of university hospital status was less clear, with the strongest associations observed for the larger cohorts and for 365-day mortality. PACU-LOS was predictive for 30-day mortality whereas no effect was seen in the cohorts with small sample sizes at 365 days (2014 and 2016). Night and weekend surgery were significantly predictive of mortality at 365-days for the 2016 cohort, but not in any of the other years, and was not related to 30-day mortality. Time of surgery, surgical waiting time, time in theatre, type of surgery were not associated with 30- or 365-day mortalities in any of the multivariable analyses, with p-values generally exceeding 0.2 (Supplementary Tables S3–8).

Since our study is more inclusive regarding age span than most previous studies on hip fracture patients (includes all adults, not only elderly people) we also re-ran the Cox regression analysis including only patients >65 years of age. This returned very similar results compared to the Cox analysis for the whole cohort.

## Discussion

In this retrospective cohort study of patients undergoing hip fracture surgery in Sweden with adjustment for patient, surgical, anaesthetic and structural factors, we identified several independent variables significantly associated with mortality. The most important associations were age, male gender, ASA-PS-class, admission to a non-university hospital and PACU-LOS. These findings were confirmed in secondary analyses using multivariable logistic regression analyses with 30-day and 365-day mortalities as the outcome.

The sensitivity analyses support the robustness of our results for age, gender and ASA-class, with similar findings when analysing each year separately. Findings for university hospital status were less robust with the largest effects seen for 365-day mortality, a finding also observed with the Kaplan-Meier analysis. In contrast, length of stay in PACU was independently associated with mortality at 30 days for all years, but the relationship at 365 days was less clear. This suggests that PACU stay has greater effects on shorter-term outcomes compared to university hospital status that has a greater effect on long-term outcomes. A visual inspection of the Kaplan-Meier curves (Figs 2 and 3) supports this suggestion. Although baseline characteristics were similar between the years, we believe that these results may be at least partially explained by differences in sample size and registry coverage that increased substantially between 2014 and 2016. University hospital status is particularly affected by registry coverage, as there were only few university hospitals in 2014. Therefore, whilst findings for PACU-LOS and university hospital status are statistically significant in the Cox analysis with narrow confidence intervals, we regard these findings as only indicative and requiring further confirmation.

The median age of this cohort was 83 (IQR 76–89) years, similar to those reported in other European databases^16,34,35^. 30- and 365-day mortality rates are also consistent with previously published registry results from other Scandinavian countries and the UK^1,16,35^. Both these figures may be considered to be unacceptably high, especially in the light of recent findings where differences in hospital mortality were found among patients who underwent hip fracture surgery compared to elective total hip replacement even after adjustment for age, sex, and preoperative comorbidities^34^. The causes of death could not be determined for this cohort as this data was not collected by this registry. However, this data may be made available by linkage to other national registries, and would be a relevant pursuit for future studies.

Patient-related factors such as age, ASA-PS-class and male gender are independently associated with mortality after hip fracture surgery which is consistent with previous studies^36–38^. Adjusted hazard ratios for patients in ASA-PS-class 3 compared to ASA 1 and 2 was 2.12 [95% CI 1.96–2.29, p < 0.001], increasing more than 4-fold for those in ASA-PS-class 4 (aHR 4.79 [95% CI 4.30–25.33, p < 0.001]), similar to the findings of a Danish study that found approximately 2-fold increases in 30-day mortality for every stratum of ASA-class^16^.

Male gender increased the risk of death by over 50%. For 30-day mortality the aHR was 1.75 (95% CI 1.53–2.01, p < 0.001) and for 365-day mortality the aHR was 1.62 (95% CI 1.48–1.70, p < 0.001), corroborating the findings in the Scottish Hip Fracture Audit and other studies^1,10,16,36–38^.

No difference was identified regarding type of anaesthesia in our multivariable models. Our results are consistent with recent retrospective cohort studies that found no differences in mortality due to anaesthetic technique^22–24^. Finally, a recent Cochrane review noted that type of anaesthesia was not a significant risk factor for 30-day mortality however the quality of evidence was low^21^. We emphasize that the present study only investigated the effect of type of anaesthesia on mortality, and that there may be other clinically relevant outcomes, such as complications, hospital length of stay, discharge destination, functional outcome and disability. The effect of anaesthetic technique therefore deserves further investigation in larger scale studies and we await the result of the on-going REGAIN study (NCT02507505).

Surgical repair within 24 hours of admission is recommended by many guidelines and surgical delay has been suggested to be an independent risk factor for mortality after hip fracture surgery. However, available data regarding the effects of delayed surgery are inconclusive. Some of this discrepancy may be due to differing definitions for ‘delayed’ where cut-offs may range from 12 to 96 hours. Another important contributor to the equipoise is differences in adjustment for confounding factors. For example, one possible clinical scenario may be an increased likelihood of delayed surgery in patients with multiple and severe medical comorbidities leading to confounding by indication, whilst some other studies do not include adjustment for comorbidities.

There is some evidence supporting the hypothesis that surgical delay is an important determinant of mortality even when adjusted for other factors, most commonly age, sex and ASA-PS-class^13,14,16,38^. In a meta-analysis, Shiga *et al*. demonstrated an adjusted OR of 1.42 (95% CI 1.36–1.47) for 30-day mortality and 1.27 (95% CI 1.12–1.44) for 365-day mortality after delayed surgery, where ‘delayed’ was defined as >48 hours after admission^13^. Daugaard *et al*. found an increased risk of in-hospital and 30-day mortality with every 24 hours of delayed surgery^16^ and a meta-analysis by Simunovic *et al*. showed a significant reduction in mortality (aRR 0.81 [CI 0.68–0.96]) if surgery was ‘early’ regardless of whether this was defined as within 24, 48 or 72 hours of admission^14^. Finally, in the only interventional study we identified, Bohm *et al*. demonstrated that a coordinated, regional intervention reduced time to surgery for hip fractures to within 48 hours of admission. This reduction in time to surgery resulted in a reduced hazard of death both in hospital (aHR 0.51 [0.41–0.63]) and at 365 days (aHR 0.72 [0.64–0.80]), after adjustment for age, sex, type of surgery and presence of comorbidities^10^.

Other studies do not support the relationship between early surgery and mortality. In a carefully conducted study with adjustment for multiple premorbid confounders and propensity matching, Orosz *et al*. did not demonstrate improved survival or function when surgery was conducted within 24 hours of admission^39^. Likewise, Grimes *et al*. showed no differences in morality in patients subjected to surgery within 24–48 h of admission compared to those where surgery was delayed beyond 96 hours^11^. In the present study, although the waiting time for surgery was associated with increased 30-and 365-day mortalities in univariate analyses, the effect was lost after adjustment for other confounders regardless of whether it was analysed as a categorical (<12 h, 12h–23:59 h and >24 h) or a continuous variable. Categorization of surgical waiting time was also done in an effort to test the effect of very early surgical intervention. We note however that the median time to surgery was 16.5 hours (IQR 8.4–22.7), well within the limits of most guidelines. Thus, we were unable to rule out an effect of very late delays. The on-going HIPATTACK study that randomizes patients to accelerated (medical clearance within 2 hours and initiation of surgery within 6 hours of the diagnosis of hip fracture) vs. standard care will provide further detail as to the potential benefits of early surgery by examining complications as the primary clinical outcome (NCT01344343)^9^.

In Sweden, a fast-track pathway for hip fracture patients has been implemented to optimize care and outcome. This fast-track may be initiated in the ambulance if a hip fracture is suspected, enabling the staff to take the patient straight to the radiology department without having to pass by the emergency department. Swedish national guidelines also recommend that hospitals should aim to get all hip fractures to surgery within 24 hours^4^. Our data indicate that has been the case in the present population with a median surgical waiting time of 16.5 hours. Moreover, national programs for care of the patient with hip fractures are widely implemented. Hence the results shown in this study must be interpreted within this context and may not be generalizable to other health care systems.

We found that type of surgery and time in theatre did not affect the mortality estimates. Surgery during evenings, nights, weekends and public holidays were not associated with increased mortality. This is in agreement with the findings of several previous studies including a Danish study using data from the Danish National Indicator Project, where population and socioeconomic characteristics may be regarded as similar to those in Sweden^15,16,20^. Other studies suggest increased mortality rates due to weekend admissions^17,40^. We believe that these discrepancies may be explained by differences in national health care systems, study design and adjustment for confounders. In general, we found little evidence in this study and in previous literature for an effect of after-hours surgery on mortality for hip fracture surgery.

The impact of PACU-LOS was significantly associated with both 30-day and 365-day mortalities, regardless of whether this was examined as a continuous variable or categorized. The evidence regarding how PACU-LOS might impact mortality is unclear although there is evidence that clinical pathways in a post-anaesthesia care unit can significantly reduce length of stay and can improve postoperative outcome^27^. In our study PACU-LOS was identified as significantly associated with mortality in the Cox regression analysis, and confirmed in the multivariable logistic regression analyses for 30- and 365-day mortalities. There may be several reasons for this. Firstly, most PACUs in Sweden provide single organ support such as vasopressor infusions, invasive haemodynamic monitoring and continuous positive airway pressure ventilation. A longer PACU-LOS may therefore be indicative of a complication or an untoward perioperative event that may have a significant impact on clinical outcome that was not controlled for in the present analyses. Although we attempted to control for comorbidity using the ASA-PS-class, this may not have adequately reflected the severity of underlying medical conditions requiring extended postoperative care. In contrast we found no relationship between ICU-admission and mortality, which we interpret cautiously. Only 4.3% of the population were admitted to ICU and we did not examine reasons for admission and advance directives for level of care.

University hospitals were significantly associated with lower mortality in the adjusted Cox regression analyses (aHR 0.83 [CI 0.76–0.91]), and in the multivariable analyses for 30- and 365-day mortalities. This finding is notable since the protective effect of university hospital status was found despite a higher mean ASA-PS-class. This is in line with the findings from 3 previous studies where careful adjustment for covariates were made^30–32^. In an analysis of 57315 hip fracture patients, Weller *et al*. found a decreased risk of death in teaching hospitals despite longer surgical delays, and a higher incidence of complications and comorbidities^32^. Similarly, a study of the National Long Term Care Survey in the United States revealed lowest mortality rates for admissions to major teaching hospitals, compared to for-profit non-teaching hospitals^31^. A great majority of hospitals in Sweden are government-run and are non-profit organisations, eliminating the profit motive as an explanation for this difference. University (=teaching) hospitals only made up 16.1% of our total study population.

We did not distinguish between university and non-university teaching hospitals, urban and rural hospitals, therefore we are unable to evaluate whether the effect seen here is due to teaching status or other hospital characteristics. We also did not examine for differences due to length of hospital stay, hospital volume, availability of rehabilitation care and specialist nursing care that may differ between university and non-university centres.

Although we attempted to account for various aspects of the perioperative process and have corrected for more covariates than most previous studies, the possibility of unmeasured confounders is almost certain. For example, we did not address socioeconomic factors, many hospital factors (eg. staffing and hospital volume), presence and severity of comorbidities (eg. using the Charlson or Elixhauser scores, premorbid cognitive impairment, polypharmacy) and postoperative factors (eg. availability of structured rehabilitation programs and discharge destination). We also only examined mortality as an end-point, but acknowledge that other end-points such as postoperative complications, discharge destination, functional status, disability, quality of recovery and patient-related outcome measures may be more relevant. We also did not examine causes of death. Although SPOR does not collect these data, linkage with other national quality registries could have provided some more information.

We did not include patients that may have died waiting for surgery or patients deemed unfit for surgery due to severe pre-existing comorbidity. However, our assessment is that this is unlikely to change the present findings.

We also acknowledge that SPOR is a growing national quality registry with a doubling in sample size between 2014 and 2016. Thus, much of the heterogeneity in the sensitivity analyses may have been due to variation in coverage. The lack of complete coverage in the registry is also a source of bias in this study.

A major strength of the present study is that it addresses a wider range of perioperative variables than most other studies which enabled us to obtain a clearer picture of factors increasing risk of death. Although most factors influencing this outcome were non-modifiable, we identified 2 possibly important modifiable factors (university hospital status and PACU-LOS) that deserve more detailed investigation in future studies. The fact that the study population consisted of all patients over 18 years who underwent hip fracture surgery in Sweden is also an advantage with respect to the generalizability of our results. No specific group within this age span were excluded for medical reasons, thus our results may be less prone to bias.

## Conclusions

Age, gender, ASA-PS-class were strong predictors of mortality after surgery for hip fractures in Sweden. University hospital status and length of stay in the post-anaesthesia care unit were also identified as important modifiable risk factors that persisted after adjustment for confounders. Whilst we are unable to attribute causality to these findings, they deserve attention in future studies, to help understand how university hospital status and postoperative care may contribute to survival.

## Electronic supplementary material

Supplementary Tables S1–8
