# Supplementary material for: Determinants of mortality after hip fracture surgery in Sweden: a registry-based retrospective cohort study
Source: Sci Rep. 2018 Oct 24;8:15695. doi: 10.1038/s41598-018-33940-8 (PMC6200788; doi:10.1038/s41598-018-33940-8)
Supplement: Supplementary file 1 — Supplementary Tables S1–8 [file 41598_2018_33940_MOESM1_ESM.docx]

**Determinants of mortality after hip fracture surgery in Sweden: a registry-based retrospective cohort study**

*Rasmus Åhman^1^

*Pontus Forsberg Siverhall^1^

Johan Snygg^2^

Mats Fredrikson^3^

Gunnar Enlund^4^

Karin Björnström ^1^

Michelle S Chew^1^

*Rasmus Åhman, Pontus Forsberg Siverhall and Johan Snygg contributed equally to this work

** Michelle S Chew and Karin Björnström are co-senior authors

^1^Department of Anaesthesia and Intensive Care, Department of Medical and Health Sciences, Linköping University, Linköping, S-581 85 Sweden

^2^Department of Anaesthesia and Intensive Care, Sahlgrenska University Hospital, 413 45

Gothenburg, Sweden

^3^Department of Clinical and Experimental Medicine, Faculty of Medicine and Health, Linköping University, S-581 85 Linköping, Sweden

^4^Department of Anaesthesia and Intensive Care, Uppsala University Hospital, 78185

Uppsala, Sweden

Supplementary Table S1

30-day mortality analysed with multivariable logistic regression.

|  | **aOR** | **95% CI** | | **p-value** |
| --- | --- | --- | --- | --- |
| Age | 1.07 | 1.06 | 1.08 | <0.001 |
| Male gender | 1.75 | 1.53 | 2.01 | <0.001 |
| ASA-PS-class 1 & 2  ASA-PS 3  ASA-PS 4  ASA-PS 5 | 2.87  7.89  20.43 | 2.39  6.28  6.38 | 3.44  9.91  65.41 | Reference  <0.001  <0.001  <0.001 |
| University hospital | 0.81 | 0.68 | 0.98 | 0.026 |
| Time of surgery |  |  |  |  |
| Day |  |  |  | Reference |
| Evening | 1.11 | 0.91 | 1.35 | 0.316 |
| Night | 0.81 | 0.35 | 1.91 | 0.635 |
| Weekend | 1.03 | 0.89 | 1.20 | 0.703 |
| Type of surgery  Osteosynthesis: cerclage, spikes, pins  Osteosynthesis: intramedullary nail  Osteosynthesis: screw and plate  Hip replacement: cemented  Hip replacement: non-cemented | 1.15  1.19  1.19  1.02 | 0.91  0.94  0.95  0.34 | 1.44  1.50  1.51  3.05 | Reference  0.243  0.140  0.133  0.977 |
| Compliance to surgical urgency planning | 0.97 | 0.71 | 1.34 | 0.869 |
| Surgical waiting time  <12h  12h-23h59min  >24h | 1.01  1.33 | 0.86  0.95 | 1.19  1.86 | Reference  0.911  0.093 |
| Time in theatre | 0.94 | 0.83 | 1.07 | 0.353 |
| Type of anaesthesia | 1.16 | 0.96 | 1.39 | 0.115 |
| PACU-LOS | 1.03 | 1.02 | 1.05 | <0.001 |
| ICU-admission | 1.03 | 0.67 | 1.60 | 0.891 |

Supplementary Table S2

365-day mortality analysed with multivariable logistic regression.

|  | **aOR** | **95% CI** | | **p-value** |
| --- | --- | --- | --- | --- |
| Age | 1.06 | 1.06 | 1.07 | <0.001 |
| Male gender | 1.62 | 1.47 | 1.77 | <0.001 |
| ASA-PS-class 1 & 2  ASA-PS 3  ASA-PS 4  ASA-PS 5 | 2.66  7.82  37.26 | 2.40  6.64  7.76 | 2.95  9.20  178.95 | Reference  <0.001  <0.001  <0.001 |
| University hospital | 0.74 | 0.65 | 0.83 | <0.001 |
| Time of surgery  Day  Evening  Night  Weekend | 1.08  1.18  1.01 | 0.94  0.72  0.92 | 1.23  1.92  1.11 | Reference  0.266  0.514  0.800 |
| Type of surgery  Osteosynthesis: cerclage, spikes, pins  Osteosynthesis: intramedullary nail  Osteosynthesis: screw and plate  Hip replacement: cemented  Hip replacement: non-cemented | 0.99  1.12  0.95  0.74 | 0.85  0.97  0.81  0.37 | 1.15  1.31  1.10  1.50 | Reference  0.867  0.127  0.470  0.403 |
| Compliance to surgical urgency planning | 1.07 | 0.86 | 1.33 | 0.550 |
| Surgical waiting time  <12h  12h-23h59min  >24h | 1.11  1.15 | 1.0  0.91 | 1.24  1.44 | Reference  0.051  0.240 |
| Time in theatre | 0.97 | 0.89 | 1.05 | 0.455 |
| Type of anaesthesia | 1.05 | 0.94 | 1.19 | 0.386 |
| PACU-LOS | 1.02 | 1.01 | 1.03 | 0.001 |
| ICU-admission | 1.17 | 0.86 | 1.59 | 0.305 |

Supplementary Table S3

Multivariable analysis for 30-day mortality, 2014 only. n=3203

|  | aOR | 95% C.I. | | p-value |
| --- | --- | --- | --- | --- |
| Age | 1.086 | 1.063 | 1.109 | <0.001 |
| Male gender | 1.593 | 1.186 | 2.140 | 0.002 |
| ASA-PS-class 1 & 2 |  |  |  | Reference |
| ASA-PS 3 | 2.490 | 1.775 | 3.493 | <0.001 |
| ASA-PS 4 | 5.973 | 3.677 | 9.703 | <0.001 |
| ASA-PS 5 | n/a | n/a | n/a | n/a |
| University Hospital | 0.733 | 0.454 | 1.183 | 0.203 |
| Time of surgery  Day |  |  |  | Reference |
| Evening | 0.931 | 0.605 | 1.433 | 0.746 |
| Night | 1.186 | 0.642 | 2.191 | 0.586 |
| Weekend | 1.135 | 0.673 | 1.914 | 0.635 |
| Type of surgery |  |  |  |  |
| Osteosynthesis: cerclage, spikes, pins |  |  |  | Reference |
| Osteosynthesis: intramedullary nail | 1.086 | 0.661 | 1.785 | 0.745 |
| Osteosynthesis: screw and plate | 1.389 | 0.838 | 2.304 | 0.203 |
| Hip replacement: cemented | 1.545 | 0.939 | 2.543 | 0.087 |
| Hip replacement: non-cemented | 1.429 | 0.169 | 12.112 | 0.743 |
| Compliance to surgical urgency planning | 1.412 | 0.718 | 2.780 | 0.317 |
| Surgical waiting time  <12h |  |  |  | Reference |
| 12h-23h59min | 1.062 | 0.748 | 1.509 | 0.735 |
| >24h | 1.424 | 0.691 | 2.934 | 0.338 |
| Time in theatre | 0.811 | 0.606 | 1.086 | 0.159 |
| Type of anaesthesia | 1.181 | 0.774 | 1.801 | 0.440 |
| PACU-LOS | 1.047 | 1.014 | 1.081 | 0.005 |
| ICU-admission | 0.723 | 0.168 | 3.123 | 0.664 |

n/a – too few samples in this category to provide adequate statistic

Supplementary Table S4

Multivariable analysis for 365-day mortality, 2014 only. n=3203

|  | aOR | 95% C.I. | | p-value |
| --- | --- | --- | --- | --- |
| Age | 1.075 | 1.061 | 1.088 | <0.001 |
| Male gender | 1.382 | 1.130 | 1.690 | 0.002 |
| ASA-PS-class 1 & 2 |  |  |  | Reference |
| ASA-PS 3 | 2.343 | 1.911 | 2.874 | <0.001 |
| ASA-PS 4 | 7.347 | 5.079 | 10.626 | <0.001 |
| ASA-PS 5 | n/a | n/a | n/a | n/a |
| University Hospital | 0.855 | 0.633 | 1.155 | 0.308 |
| Time of surgery  Day |  |  |  | Reference |
| Evening | 1.209 | 0.908 | 1.609 | 0.193 |
| Night | 1.144 | 0.767 | 1.707 | 0.509 |
| Weekend | 1.078 | 0.772 | 1.504 | 0.660 |
| Type of surgery |  |  |  |  |
| Osteosynthesis: cerclage, pikes, pins |  |  |  | Reference |
| Osteosynthesis: intramedullary nail | 0.903 | 0.658 | 1.240 | 0.529 |
| Osteosynthesis: screw and plate | 1.010 | 0.729 | 1.398 | 0.954 |
| Hip replacement: cemented | 0.930 | 0.671 | 1.287 | 0.660 |
| Hip replacement: non-cemented | 0.219 | 0.027 | 1.769 | 0.154 |
| Compliance to surgical urgency planning | 1.203 | 0.778 | 1.860 | 0.406 |
| Surgical waiting time  <12h |  |  |  | Reference |
| 12h-23h59min | 1.257 | 0.995 | 1.587 | 0.055 |
| >24h | 1.519 | 0.951 | 2.426 | 0.080 |
| Time in theatre | 0.986 | 0.824 | 1.180 | 0.879 |
| Type of anaesthesia | 0.891 | 0.686 | 1.158 | 0.390 |
| PACU-LOS | 1.023 | 0.998 | 1.049 | 0.071 |
| ICU-admission | 0.599 | 0.201 | 1.784 | 0.358 |

n/a – too few samples in this category to provide adequate statistic

Supplementary Table S5

Multivariable analysis for 30-day mortality, 2015 only. n= 5187

|  | aOR | 95% C.I. | | p-value |
| --- | --- | --- | --- | --- |
| Age | 1.067 | 1.051 | 1.084 | <0.001 |
| Male gender | 1.948 | 1.543 | 2.459 | <0.001 |
| ASA-PS-class 1 & 2 |  |  |  | Reference |
| ASA-PS 3 | 3.045 | 2.211 | 4.192 | <0.001 |
| ASA-PS 4 | 8.564 | 5.722 | 12.816 | <0.001 |
| ASA-PS 5 | 7.190 | 0.576 | 89.786 | 0.126 |
| University Hospital | 0.715 | 0.509 | 1.005 | 0.053 |
| Time of surgery  Day |  |  |  | Reference |
| Evening | 1.011 | 0.722 | 1.414 | 0.951 |
| Night | 0.980 | 0.598 | 1.605 | 0.935 |
| Weekend | 0.933 | 0.622 | 1.400 | 0.738 |
| Type of surgery |  |  |  |  |
| Osteosynthesis: cerclage, spikes, pins |  |  |  | Reference |
| Osteosynthesis: intramedullary nail | 1.079 | 0.728 | 1.598 | 0.705 |
| Osteosynthesis: screw and plate | 1.033 | 0.697 | 1.531 | 0.872 |
| Hip replacement: cemented | 1.065 | 0.718 | 1.580 | 0.754 |
| Hip replacement: non-cemented | 1.277 | 0.280 | 5.838 | 0.752 |
| Compliance to surgical urgency planning | 0.961 | 0.574 | 1.610 | 0.881 |
| Surgical waiting time  <12h |  |  |  | Reference |
| 12h-23h59min | 0.937 | 0.706 | 1.243 | 0.652 |
| >24h | 1.298 | 0.752 | 2.238 | 0.349 |
| Time in theatre | 0.983 | 0.793 | 1.220 | 0.879 |
| Type of anaesthesia | 1.083 | 0.796 | 1.473 | 0.612 |
| PACU-LOS | 1.052 | 1.023 | 1.081 | <0.001 |
| ICU-admission | 0.922 | 0.491 | 1.733 | 0.802 |

n/a – too few samples in this category to provide adequate statistic

Supplementary Table S6

Multivariable analysis for 365-day mortality, 2015 only. n=5187

|  | aOR | 95% C.I. | | p-value |
| --- | --- | --- | --- | --- |
| Age | 1.062 | 1.052 | 1.072 | <0.001 |
| Male gender | 1.734 | 1.483 | 2.027 | <0.001 |
| ASA-PS-class 1 & 2 |  |  |  | Reference |
| ASA-PS 3 | 2.776 | 2.331 | 3.305 | <0.001 |
| ASA-PS 4 | 10.206 | 7.617 | 13.675 | <0.001 |
| ASA-PS 5 | n/a | n/a | n/a | n/a |
| University Hospital | 0.670 | 0.537 | 0.835 | <0.001 |
| Time of surgery  Day |  |  |  | Reference |
| Evening | 1.002 | 0.805 | 1.247 | 0.988 |
| Night | 0.967 | 0.701 | 1.334 | 0.839 |
| Weekend | 0.973 | 0.745 | 1.271 | 0.841 |
| Type of surgery |  |  |  |  |
| Osteosynthesis: cerclage, spikes, pins |  |  |  | Reference |
| Osteosynthesis: intramedullary nail | 0.906 | 0.704 | 1.166 | 0.445 |
| Osteosynthesis: screw and plate | 1.010 | 0.786 | 1.298 | 0.938 |
| Hip replacement: cemented | 0.838 | 0.65 | 1.078 | 0.169 |
| Hip replacement: non-cemented | 0.964 | 0.369 | 2.518 | 0.941 |
| Compliance to surgical urgency planning | 0.862 | 0.609 | 1.220 | 0.401 |
| Surgical waiting time  <12h |  |  |  | Reference |
| 12h-23h59min | 1.034 | 0.863 | 1.237 | 0.718 |
| >24h | 1.046 | 0.723 | 1.514 | 0.810 |
| Time in theatre | 1.068 | 0.931 | 1.225 | 0.349 |
| Type of anaesthesia | 0.963 | 0.789 | 1.177 | 0.715 |
| PACU-LOS | 1.028 | 1.006 | 1.050 | 0.012 |
| ICU-admission | 0.971 | 0.627 | 1.502 | 0.894 |

n/a – too few samples in this category to provide adequate statistic

Supplementary Table S7

Multivariable analysis for 30-day mortality, 2016 only. n= 6542

|  | aOR | 95% C.I. | | p-value |
| --- | --- | --- | --- | --- |
| Age | 1.073 | 1.058 | 1.088 | <0.001 |
| Male gender | 1.693 | 1.377 | 2.082 | <0.001 |
| ASA-PS-class 1 & 2 |  |  |  | Reference |
| ASA-PS 3 | 3.115 | 2.307 | 4.205 | <0.001 |
| ASA-PS 4 | 8.937 | 6.265 | 12.749 | <0.001 |
| ASA-PS 5 | n/a | n/a | n/a | n/a |
| University Hospital | 0.923 | 0.721 | 1.180 | 0.522 |
| Time of surgery  Day |  |  |  | Reference |
| Evening | 1.253 | 0.930 | 1.687 | 0.138 |
| Night | 1.082 | 0.700 | 1.672 | 0.724 |
| Weekend | 1.198 | 0.928 | 1.547 | 0.165 |
| Type of surgery |  |  |  |  |
| Osteosynthesis: cerclage, spikes, pins |  |  |  | Reference |
| Osteosynthesis: intramedullary nail | 1.219 | 0.865 | 1.718 | 0.258 |
| Osteosynthesis: screw and plate | 1.274 | 0.895 | 1.814 | 0.179 |
| Hip replacement: cemented | 1.170 | 0.820 | 1.668 | 0.387 |
| Hip replacement: non-cemented | 0.485 | 0.047 | 5.011 | 0.543 |
| Compliance to surgical urgency planning | 0.906 | 0.539 | 1.525 | 0.711 |
| Surgical waiting time  <12h |  |  |  | Reference |
| 12h-23h59min | 1.057 | 0.822 | 1.361 | 0.666 |
| >24h | 1.269 | 0.740 | 2.175 | 0.386 |
| Time in theatre | 0.949 | 0.778 | 1.158 | 0.607 |
| Type of anaesthesia | 1.207 | 0.921 | 1.583 | 0.173 |
| PACU-LOS | 1.025 | 1.009 | 1.041 | 0.002 |
| ICU-admission | 1.302 | 0.663 | 2.554 | 0.443 |

n/a – too few samples in this category to provide adequate statistic

Supplementary Table S8

Multivariable analysis for 365-day mortality, 2016 only. n=1965

|  | aOR | 95% C.I. | | p-value |
| --- | --- | --- | --- | --- |
| Age | 1.062 | 1.046 | 1.078 | <0.001 |
| Male gender | 1.832 | 1.424 | 2.356 | <0.001 |
| ASA-PS-class 1 & 2 |  |  |  | Reference |
| ASA-PS 3 | 2.935 | 2.184 | 3.944 | <0.001 |
| ASA-PS 4 | 8.404 | 5.384 | 13.120 | <0.001 |
| ASA-PS 5 | n/a | n/a | n/a | n/a |
| University Hospital | 0.653 | 0.468 | 0.909 | 0.012 |
| Time of surgery  Day |  |  |  | Reference |
| Evening | 1.052 | 0.728 | 1.521 | 0.787 |
| Night | 1.745 | 1.028 | 2.962 | 0.039 |
| Weekend | 1.766 | 1.115 | 2.799 | 0.015 |
| Type of surgery |  |  |  |  |
| Osteosynthesis: cerclage, spikes, pins |  |  |  | Reference |
| Osteosynthesis: intramedullary nail | 0.808 | 0.543 | 1.201 | 0.291 |
| Osteosynthesis: screw and plate | 1.007 | 0.675 | 1.502 | 0.973 |
| Hip replacement: cemented | 0.735 | 0.491 | 1.102 | 0.136 |
| Hip replacement: non-cemented | n/a | n/a | n/a | n/a |
| Compliance to surgical urgency planning | 0.891 | 0.473 | 1.677 | 0.721 |
| Surgical waiting time  <12h |  |  |  | Reference |
| 12h-23h59min | 1.033 | 0.768 | 1.389 | 0.829 |
| >24h | 0.973 | 0.498 | 1.901 | 0.936 |
| Time in theatre | 0.802 | 0.623 | 1.032 | 0.086 |
| Type of anaesthesia | 1.343 | 0.962 | 1.874 | 0.083 |
| PACU-LOS | 1.009 | 0.986 | 1.033 | 0.445 |
| ICU-admission | 1.224 | 0.638 | 2.348 | 0.544 |

n/a – too few samples in this category to provide adequate statistic
